# Fatal Multiorgan Failure Associated with Disseminated Herpes Simplex Virus-1 Infection: A Case Report

**DOI:** 10.1155/2012/359360

**Published:** 2012-09-06

**Authors:** Michael Glas, Sigrun Smola, Thorsten Pfuhl, Juliane Pokorny, Rainer M. Bohle, Arno Bücker, Jörn Kamradt, Thomas Volk

**Affiliations:** ^1^Department of Anesthesiology, Intensive Care and Pain Therapy, Saarland University Hospital, Kirrberger Straße, D-66421 Homburg, Germany; ^2^Institute of Virology, Saarland University Hospital, D-66421 Homburg, Germany; ^3^Institute of Pathology, Saarland University Hospital, D-66421 Homburg, Germany; ^4^Department of Diagnostic and Interventional Radiology, Saarland University Hospital, D-66421 Homburg, Germany; ^5^Department of Urology and Pediatric Urology, Saarland University Hospital, D-66421 Homburg, Germany

## Abstract

Herpes simplex virus type 1 (HSV-1) infections cause typical dermal and mucosal lesions in children and adults. Also complications to the peripheral and central nervous system, pneumonia or hepatitis are well known. However, dissemination to viscera in adults is rare and predominantly observed in immunocompromised patients. Here we describe the case of a 70-year-old male admitted with macrohematuria and signs of acute infection and finally deceasing in a septic shock with multi organ failure 17 days after admission to intensive care unit. No bacterial or fungal infection could be detected during his stay, but only two days before death the patient showed signs of rectal, orolabial and genital herpes infection. The presence of HSV-1 was detected in swabs taken from the lesions, oropharyngeal fluid as well as in plasma. Post-mortem polymerase chain reaction analyses confirmed a disseminated infection with HSV-1 involving various organs and tissues but excluding the central nervous system. Autopsy revealed a predominantly retroperitoneal diffuse large B-cell lymphoma as the suspected origin of immunosuppression underlying herpes simplex dissemination.

## 1. Introduction

Newly acquired or reactivated infections of HSV-1 cause a typical vesicular rush with orolabial ulcerations, gingivostomatitis, keratitis, and also genital/perianal affections in children and adults. Complications to the peripheral and central nervous system (meningitis, encephalitis), pneumonia, or hepatitis are well known [1–3]. Viraemia and dissemination to viscera in adults are rare and can predominantly be seen in immunocompromised individuals like patients with hemato-oncologic malignancies, transplant recipients, or due to immunosuppressive medication [4, 5]. Here we describe a case of fulminant septic shock in a patient associated with a disseminated infection with HSV-1.

## 2. Case Presentation

A 70-year-old male patient was initially admitted to a primary hospital with fever, productive cough, fatigue, reduced vigilance, and macrohematuria. His past medical history included obesity (body mass index 38.1 kg/m^2^), arterial hypertension, obstructive sleep apnea, benign prostate hyperplasia, complete atrioventricular block (implanted dual chamber pacemaker), chronic heart failure, and atrial fibrillation. For the latter, the patient was orally anticoagulated with phenprocoumon. Due to chronic backache, he received lumbar injections at regular intervals. A few weeks prior to the present admission, he travelled to Tunisia and Turkey. HIV and hepatitis B/C virus serology results were negative. Blood chemistry showed elevated C-reactive protein (219 mg/L), leukocytosis (14.2 per nL), an increase in procalcitonin (0.507 ng/mL), an international normalized ratio of 5.2, and an increased creatinine (5.0 mg/dL). Searching for a septic focus an abdominal computed tomography (CT) showed massive bilateral hydronephrosis with ureteral compression by a retroperitoneal space-occupying lesion suspected to be a hematoma causing urinary retention and urosepsis. 

**Figure 1:**
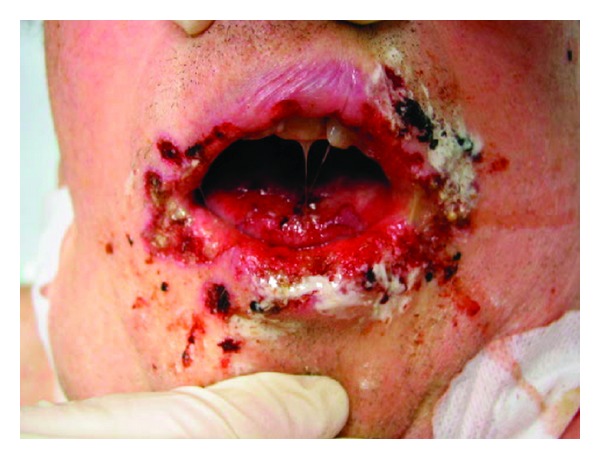
Orolabial vesicles and ulcers. Appearance of orolabial vesicles and ulcers on day 17 of ICU treatment, two days before death (percutaneous tracheotomy was performed on day 7).

For technical reasons, only the left ureter was stented with a double-j catheter. Calculated antibiotic treatment with Meropenem/Ciprofloxacin was initiated, and the patient was subsequently transferred to our hospital. Shortly after relieving the right ureter his state rapidly deteriorated. Body temperature increased to 39.9°C. His oxygenation index (136) necessitated invasive ventilation (fraction of inspired oxygen 0.6, positive end-expiratory pressure 12 mbar), and his hemodynamic status had to be supported by norepinephrine (up to 1.0 *μ*g/kg/min). On day five, he became anuric necessitating continuous venovenous hemodialysis. Surgical exploration of the right retroperitoneal space was performed to exclude an infected hematoma and revealed no relevant bleeding. Microbiological analysis (blood cultures) did not show any systemic bacterial or fungal infections, merely urine culture was initially positive for candida albicans. Elevated transaminases and increased international normalized ratio, despite substitution of prothrombin complex, indicated the beginning of liver failure. Although procalcitonin fluctuated between 2.2 and a maximum of 7.3 ng/mL, repeated blood culture results did not show bacteremia or candidemia. Reevaluating a septic focus with CT scans (cranial, thoracic, and abdominal) between intensive care unit days 8 and 16 still showed the retroperitoneal space-occupying lesion without signs of inflammation. Surgical exploration of the right retroperitoneum on day 16 did not find any correlate. On day 17, colorectal endoscopy revealed several widespread rectal ulcers (up to 25 mm in diameter)—with suspect of viral origin. On the same day (almost four weeks after admission to hospital) oral aphtous ulcers, orolabial, and genital herpetiform vesicles (Figure 1) appeared. Samples obtained from oral and genital swabs as well as oropharyngeal fluid were analyzed by (semiquantitative) real-time polymerase chain reaction (PCR, LightCycler System, Roche, Basel, Switzerland) and revealed HSV-1 DNA in all samples. In addition, HSV-1 was detected in EDTA (ethylenediaminetetraacetic acid) plasma samples indicating florid systemic infection. EDTA blood and oropharyngeal fluid were additionally positive for Epstein-Barr virus, with 2,400 copies per mL and 27,000 copies per mL, respectively. Copy numbers for cytomegalovirus, varicella-zoster virus, and BK virus (human polyoma virus 1) were below the detection limit. Antiviral therapy (acyclovir, adapted to renal failure and dialysis) was immediately initiated. Two days later the patient died of multiple organ failure and septic shock on ICU day 19 (four weeks after admission to primary hospital).

**Figure 2:**
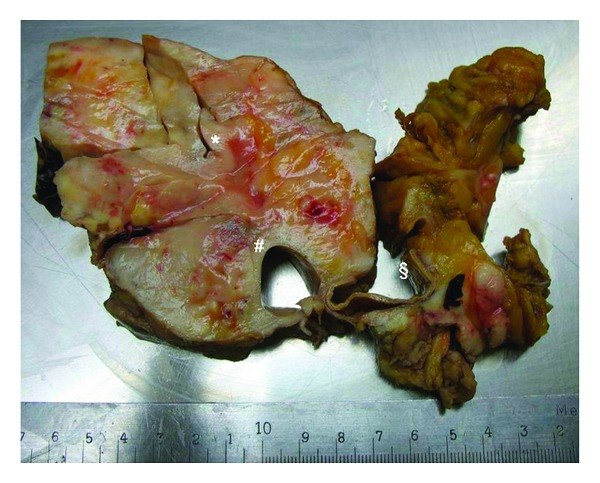
Autopsy revealed a massive retroperitoneal lymphoma. Giant retroperitoneal lymphoma masses enclosing abdominal aorta [§], inferior vena cava [#] and leading to stenosis of the right ureter [∗]. Tissue was formalin fixed.

On autopsy the retroperitoneal space-occupying lesion revealed a solid tumor (21 × 18 × 9.5 cm) expanding from the renal artery to the common right iliac artery and right psoas muscle, enclosing the inferior vena cava and the abdominal aorta (see Figure 2), leading to ureter stenosis and dilatation on both sides as well as to infiltration of the bladder. Microscopic examination and immunohistochemical analyses finally showed a diffuse of large B-cell lymphoma. As there was further tumor infiltration of the mesenterial root (15 × 6 × 4.8 cm), pericardium (up to 5 × 4 × 3.2 cm), cervical lymph nodes (3 cm in diameter), right visceral pleura (inferior and middle lobe), serosa of the colon, and periadrenal fat tissue, the lymphoma was categorized as Ann-Arbor stage four. Autopsy could not provide evidence for a retroperitoneal hematoma. Semiquantitative real-time PCR of postmortem specimens showed disseminated infection with HSV-1 of a variety of organs including heart, respiratory system, liver, spleen, kidney, intestines, lymphatic nodes, and muscle except for cerebrum and cerebellum (see Tables 1 and 2). Highest numbers of genome copies could be detected in organs of the gastrointestinal tract as well as in upper and lower respiratory system. 

## 3. Discussion

In developed countries the incidence of viral sepsis is believed to be less than 1% of septic episodes of patients admitted to intensive care [6]. Cases of herpes simplex dissemination leading to fulminant (multi) organ failure by hepatitis [7, 8], pneumonia [9], or even sepsis [10] are well described for immunocompetent and immunocompromised individuals including patients with hematologic malignancies [11]. 

Although there are demographic differences, seroprevalence for HSV-1 in adults is greater than 50% in Europe and infection usually takes place in the first two decades [12, 13]. Reactivation of the latent infection can be asymptomatic (recrudescence) or symptomatic (recurrence) and is supposed to be triggered by stress, hormonal changes, and immunosuppression [13, 14]. 

While leukopenia has been reported in 43% of cases with HSV-associated hepatic failure, accompanied by thrombocytopenia (45%), and elevation of transaminases and bilirubin [15, 16], our patient showed leukocytosis in a range from 14.2/nL to 26.6/nL and signs of disseminated intravascular coagulation. Increased procalcitonin values correlated with the conditions of a septic inflammatory response syndrome and multiorgan failure. Although procalcitonin levels are frequently below 0.5 ng/mL in viral infections, values up to 17 ng/mL and an overlap with severe bacterial infection were described for systemic viral infections [17]. The spectrum of symptoms of the disseminated HSV infection resembles the clinical picture of a bacterial sepsis, which is reflected by analogies in inflammatory host response.

The relevance of HSV-1 detection in distinct body compartments and the impact of antiviral treatment for patients' outcome are still a matter of debate. In a recent study, Berrington et al. examined the clinical correlates of HSV-1/2 in 951 serum or plasma samples. 4% of those patients had detectable levels of HSV-1/2 in PCR analysis and were observed to have a high mortality rate [18]. In this course, a review of 13 patients' medical records identified sepsis and multiorgan failure as the most common causes of death in immunosuppressed as well as immunocompetent individuals. Moreover, the detection of HSV-1 in bronchoalveolar lavage fluid (BALF) was shown to be related to poor outcome in ICU patients by Linssen et al. [19]. HSV-1 genome was detected in 32% of BALF samples from ICU patients compared to 15% from non-ICU patients. In this study, ICU treatment and age over 50 years were significantly associated with HSV-1 in BALF. Detection of more than 10^5^ genome copies/mL BALF was an independent predictor and reflected an increased mortality rate of 21% in critically ill patients. In contrast, Scheithauer et al. reported 191 patients with pulmonary diseases suspected to be of viral origin with 32.5% of the respiratory specimens tested positive for HSV-1 by PCR [20]. In this context, no significant differences with respect to incidence of renal insufficiency, markers of inflammation, sepsis, need for catecholamines, and mortality were found between HSV-1-positive and -negative individuals except for days of mechanical ventilation. It is of note that treatment with acyclovir did not significantly influence mortality (*P* = 0.26) although viral load in plasma decreased significantly [20]. Referring to antiviral therapy no details were mentioned about the starting point of acyclovir administration. 

In our patient, the primary hypothesis of an infected retroperitoneal hematoma had to be reassessed when his condition further decreased after sufficient drainage of the kidneys by ureteral catheters and while surgical retroperitoneal exploration and microbiological diagnostic finally excluded a superinfected retroperitoneal hematoma. Although PCR results revealed a florid systemic HSV infection, before mortem; antiviral therapy could not stem the tide. 

In retrospect, it needs to be discussed why the lymphoma was not recognized during the surgical explorations. First, based on the diagnosis of a superinfected retroperitoneal hematoma, an extraperitoneal flank incision was chosen to minimize the risk of contaminating the peritoneal cavity. Although the retroperitoneum was inspected with this approach, the solid masses were hidden in the plenteous fatty tissue of the obese patient. Second, the surgical exploration on day 16 was misguided by the hypothesis of an inflammatory process in the retroperitoneum causing the worsening septic condition of the patient. No abscess was found, and diffuse bleeding in the retroperitoneum during surgery prohibited further extensive exploration.

Having repeatedly negative blood cultures for bacterial and fungal pathogens, we assume that the presence of a diffuse large B-cell lymphoma going along with the dissemination of HSV finally caused multiorgan failure in the present case. However, it remains unclear whether the presence of herpes simplex virus in blood only acted as an indicator for a disturbed immune function caused by a terminal malignant disease, or whether disseminated HSV infection was the origin of sepsis and multiorgan failure. 

The importance of systemic HSV analysis and viral load determination in immunocompromised as well as in immunocompetent ICU patients and the necessity of prophylactic or preemptive therapy in this setting have to be clarified in future studies.

## Figures and Tables

**Table 1:** Results of the semiquantitative PCR performed two days before death. The *c*_T_-value (cycle threshold) specifies the number of cycles till the beginning of exponential phase when sample fluorescence exceeds background fluorescence for the first time. Low *c*_T_-values correlate with high copy numbers of HSV-1 DNA.

Tissue	Herpes simplex 1 DNA (qualitative)	*c*_T_-values in RT-PCR
EDTA blood	+	36
Oropharyngeal fluid	++++	17
Trial smear test	++++	20
Perianal smear test	++	33

**Table 2:** Postmortem detection of HSV-1 DNA in various tissues. Results of the semiquantitative PCR performed after mortem detecting virus DNA in all tested tissues except for cerebrum and cerebellum (*c*_T_-value: cycle threshold).

Tissue	Herpes simplex virus-1 DNA (qualitative)	*c*_T_-values in RT-PCR
Spleen	++	32
Liver	++	30
Left Kidney	++	30
Retroperitoneal lymphoma	++	33
Lymph nodes	++	33
Bone marrow	+	36
Muscle	++	33
Cerebrum	—	—
Cerebellum	—	—
Oral mucosa	++++	24
Trachea	++++	23
Right main bronchus	++++	23
Right lung (lower lobe)	+++	28
Right lung (upper lobe)	++	31
Right ventricle	+++	26
Left ventricle	++	30
Stomach	+++	28
Small intestine	+++	27
Colon	++	34

## References

[1] James E, Robinson L, Griffiths PD, Prentice HG (1996). Acute myeloblastic leukaemia presenting with herpes simplex type-1 viraemia and pneumonia. *British Journal of Haematology*.

[2] Olson LC, Buescher EL, Artenstein MS, Parkman PD (1967). Herpesvirus infections of the human central nervous system. *New England Journal of Medicine*.

[3] Plastiras S, Kampessi O (2009). Acute lymphocytic crisis following herpes simplex type 1 virus hepatitis in a nonimmunocompromised man: a case report. *Journal of Medical Case Reports*.

[4] Khera P, Haught JM, McSorley J, English JC (2009). Atypical presentations of herpesvirus infections in patients with chronic lymphocytic leukemia. *Journal of the American Academy of Dermatology*.

[5] Zuckerman R, Wald A (2009). Herpes simplex virus infections in solid organ transplant recipients. *American Journal of Transplantation*.

[6] Vincent JL, Sakr Y, Sprung CL (2006). Sepsis in European intensive care units: results of the SOAP study. *Critical Care Medicine*.

[7] Wesley Farr R, Short S, Weissman D (1997). Fulminant hepatitis during herpes simplex virus infection in apparently immunocompetent adults: report of two cases and review of the literature. *Clinical Infectious Diseases*.

[8] Fahy RJ, Crouser E, Pacht ER (2000). Herpes simplex type 2 causing fulminant hepatic failure. *Southern Medical Journal*.

[9] Prellner T, Flamholc L, Haidl S, Lindholm K, Widell A (1992). Herpes simplex virus—the most frequently isolated pathogen in the lungs of patients with severe respiratory distress. *Scandinavian Journal of Infectious Diseases*.

[10] Zahariadis G, Jerome KR, Corey L (2003). Herpes simplex virus-associated sepsis in a previously infected immunocompetent adult. *Annals of Internal Medicine*.

[11] Muller SA, Herrmann EC, Winkelmann RK (1972). Herpes simplex infections in hematologic malignancies. *The American Journal of Medicine*.

[12] Pebody RG, Andrews N, Brown D (2004). The seroepidemiology of herpes simplex virus type 1 and 2 in Europe. *Sexually Transmitted Infections*.

[13] Steiner I, Kennedy PG, Pachner AR (2007). The neurotropic herpes viruses: herpes simplex and varicella-zoster. *The Lancet Neurology*.

[14] Alleyne SA, Bukhari SS, Fraser M, Spiers P (2010). Extensive perinephric abscess complicated by herpes simplex virus 1 reactivation. *Journal of Clinical Pathology*.

[15] Sevilla J, Fernández-Plaza S, González-Vicent M (2004). Fatal hepatic failure secondary to acute herpes simplex virus infection. *Journal of Pediatric Hematology/Oncology*.

[16] Biancofiore G, Bisà M, Bindi LM (2007). Liver transplantation due to Herpes Simplex virus-related sepsis causing massive hepatic necrosis after thoracoscopic thymectomy. *Minerva Anestesiologica*.

[17] Assicot M, Gendrel D, Carsin H, Raymond J, Guilbaud J, Bohuon C (1993). High serum procalcitonin concentrations in patients with sepsis and infection. *The Lancet*.

[18] Berrington WR, Jerome KR, Cook L, Wald A, Corey L, Casper C (2009). Clinical correlates of herpes simplex virus viremia among hospitalized adults. *Clinical Infectious Diseases*.

[19] Linssen CF, Jacobs JA, Stelma FF (2008). Herpes simplex virus load in bronchoalveolar lavage fluid is related to poor outcome in critically ill patients. *Intensive care medicine*.

[20] Scheithauer S, Manemann AK, Krüger S (2010). Impact of herpes simplex virus detection in respiratory specimens of patients with suspected viral pneumonia. *Infection*.

